# Spin-resolved band structure of heterojunction Bi-bilayer/3D topological insulator in the quantum dimension regime in annealed Bi_2_Te_2.4_Se_0.6_

**DOI:** 10.1038/srep45797

**Published:** 2017-04-05

**Authors:** I. I. Klimovskikh, D. Sostina, A. Petukhov, A. G. Rybkin, S. V. Eremeev, E. V. Chulkov, O. E. Tereshchenko, K. A. Kokh, A. M. Shikin

**Affiliations:** 1 Saint Petersburg State University, 198504, Saint Petersburg, Russia; 2 Institute of Strength Physics and Materials Science, 634055, Tomsk, Russia; 3 Tomsk State University, 634050, Tomsk, Russia; 4 Donostia International Physics Center (DIPC), 20018 San Sebastián/Donostia, Basque Country, Spain; 5 Departamento de Física de Materiales UPV/EHU, Centro de Física de Materiales CFM - MPC and Centro Mixto CSIC-UPV/EHU, 20080 San Sebastián/Donostia, Basque Country, Spain; 6 A.V. Rzhanov Institute of Semiconductor Physics, 630090, Novosibirsk, Russia; 7 Novosibirsk State University, 630090, Novosibirsk, Russia; 8 V.S. Sobolev Institute of Geology and Mineralogy, 630090, Novosibirsk, Russia

## Abstract

Two- and three-dimensional topological insulators are the key materials for the future nanoelectronic and spintronic devices and quantum computers. By means of angle- and spin-resolved photoemission spectroscopy we study the electronic and spin structure of the Bi-bilayer/3D topological insulator in quantum tunneling regime formed under the short annealing of Bi_2_Te_2.4_Se_0.6_. Owing to the temperature-induced restructuring of the topological insulator’s surface quintuple layers, the hole-like spin-split Bi-bilayer bands and the parabolic electronic-like state are observed instead of the Dirac cone. Scanning Tunneling Microscopy and X-ray Photoemission Spectroscopy measurements reveal the appearance of the Bi_2_ terraces at the surface under the annealing. The experimental results are supported by density functional theory calculations, predicting the spin-polarized Bi-bilayer bands interacting with the quintuple-layers-derived states. Such an easily formed heterostructure promises exciting applications in spin transport devices and low-energy electronics.

Topological insulator (TI) phase, realized in materials with strong spin-orbit interaction, became one of the most intriguing topics in condensed matter physics. TIs are bulk band insulators but have the metallic Dirac cone-like edge states protected by time-reversal symmetry. The conception of TIs can be realized in three-dimensional (3D) and two-dimensional (2D) materials^1^,^2^. The key feature of the topological surface/edge states is the spin-momentum locking that is extremely useful in relation to possible applications in spintronics and nanoelectronics^1^,^3^,^4^,^5^,^6^,^7^. Noteworthy, 2D TIs have an advantage over 3D TIs in spin transport devices because of the two well-defined directions for moving of electrons via the 1D metallic edge states^8^,^9^. Additionally, the contact of 2D and 3D TIs is of particular interest owing to the interplay of 2D and 1D spin transport channels. Such topological heterostructures have been grown and studied in several systems^10^,^11^,^12^,^13^,^14^ including graphene^15^,^16^,^17^, which can be turned to topological phase via contact with heavy atoms^18^.

Recently a single-bilayer Bi (111) ultrathin film has been revealed as the most promising 2D TI, since it has the well localized edge states in contrast to the HgTe quantum well^19^,^20^,^21^. Furthermore, the Bi-bilayer contacted with 3D TI in the quantum dimension regime has recently been proposed to be a platform for topological edge mode-based transistor^21^. The crystal structure and the in-plane lattice constant is similar for Bi(111) and most of 3D TIs (e.g. Bi_2_Te_3_). It allows to grow the epitaxial 2D/3D TIs heterostructures and electronic and spin properties of MBE grown Bi bilayer on 3D TIs have been investigated^10^,^11^,^12^,^13^,^22^,^23^,^24^. For the fabrication purposes, the relatively easier way to produce the Bi bilayer film on bismuth chalcogenides by the sample annealing have recently been developed in refs 25 and 26. By means of STM authors have measured detailed surface morphology and local density of states and revealed the Bi bilayer formation at the surface of Bi_2_Te_3_ as a result of annealing. However, to unveil the topology of the surface and interface states direct electronic and spin structure measurements are required.

In this work, we study the surface restructuring of 3D topological insulator Bi_2_Te_2.4_Se_0.6_ (BTS) under the brief annealing. Scanning tunneling microscopy (STM) and X-ray Photoemission Spectroscopy (XPS) measurements reveal the transformation of the surface quintuple layers (QLs) and appearance of Bi-bilayer terraces as a result of the annealing of the sample up to 400 °C. By means of Spin- and Angle-Resolved Photoemission Spectroscopy (SARPES) we study the alteration of the Dirac cone-like electronic structure near the Fermi level of the annealed BTS. Owing to the evaporation of the Te and Se atoms at the surface, the Dirac cone-like surface state is replaced by the parabolic electron-like band derived from non-destroyed QLs and hole-like Bi-bilayer bands. DFT calculations and spin resolved spectra show the spin polarization of these states, and the effects of hybridization between them.

## Results and Discussion

Most of the 3D topological insulators, such as bismuth chalcogenides, are characterized by the layered crystal structure^1^,^27^. Thus, Bi_2_Te_3_ consists of Bi and Te atomic layers, stacked to each other, forming quintuple layer blocks (QLs) separated by the van der Waals spacing. However, the Bi_2_Te_3_ and related binary TIs are naturally *n*-type compounds with the Fermi level lying in the bulk conduction band. An addition of the third component and using of the fractional stoichiometry leads to the shifting of the Dirac point and tuning of the Fermi level inside the bulk energy gap. In refs 27 and 28 it has been shown that the compound Bi_2_Te_2.4_Se_0.6_ (BTS) exhibits the pronounced Dirac cone-like topological surface states (TSS), with the Dirac point located at the binding energy of 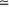
0.3 eV. In this system the Se atoms substitute the Te atoms preferably at the middle layer of QL.

**Figure 1:**
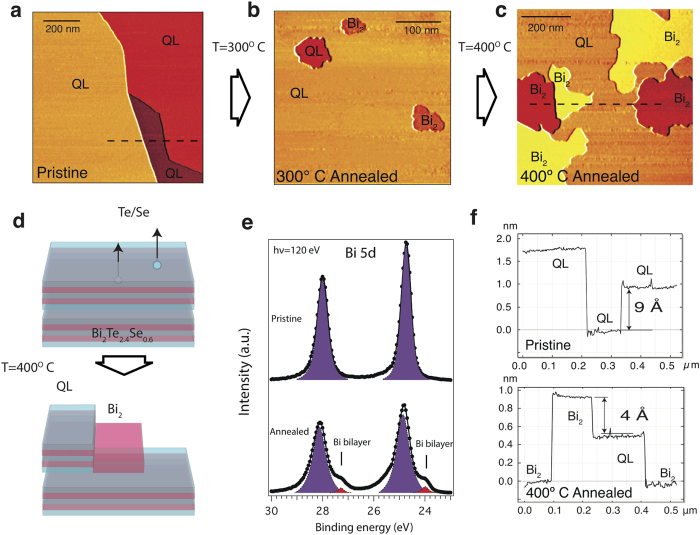
(**a**) Constant-current STM images of the cleaved pristine surface of Bi_2_Te_2.4_Se_0.6_ (set point values *V* = +1.32 V, *I* = 5 pA), (**b**) 300 °C annealed sample (*V* = +1.34 V, *I* = 10 pA) and (**c**) 400 °C annealed sample (*V* = +1.34 V, *I* = 5 pA). (**d**) Scheme of the Bi-bilayer formation at the surface of Bi_2_Te_2.4_Se_0.6_ under the annealing. Bi and Te/Se layers are shown by red and blue colors, respectively. (**e**) XPS Bi 5d spectra of the cleaved pristine (top) and annealed at 400 °C Bi_2_Te_2.4_Se_0.6_ (bottom). Data obtained with a photon energy of 120 eV at the normal emission. (**f**) Height profile along the blue line in (**c**) revealing the presence of the terraces with different step height due to the Bi-bilayer formation.

Figure 1a presents the STM image of the pristine fresh cleaved Bi_2_Te_2.4_Se_0.6_ sample. Three large terraces with the steps of 9 Å height are clearly seen, in accordance with the STM measurements of the similar bismuth chalcogenides systems, where typical thickness of QL is of 9–10 Å.

At the surface this quintuple-layer structure can be strongly modified by the short annealing of the sample. As noted above, the temperature-induced formation of the Bi-bilayer had been shown at the surface of Bi_2_Te_3_ in ref. 25. Possible explanation of the structural modification is the thermal desorption of Te atoms. Similar behavior with a Se atoms desorption have been observed in Bi_2_Se_3_^29^. Moreover, according to the phase diagrams, at temperatures of 300–400 °C the Bi-rich compounds, such as Bi_4_Te_3_ are more stable^30^,^31^.

Structural properties of Bi_2_Te_2.4_Se_0.6_ are the same as for Bi_2_Te_3_ and thus, the thermal-induced Bi-bilayer formation can also be expected. The STM image of the Bi_2_Te_2.4_Se_0.6_ surface after the annealing at 300 °C is shown in Fig. 1b. It’s seen that in addition to the large quintuple-layer terraces the holes with the depth of 6 Å appear due to evaporation of Te and Se atoms. The holes with the depth of 9 Å are also visible, that corresponds to the whole quintuple-layer removal. After the annealing at 400 °C the Bi-bilayer terraces become larger and more pronounced, as seen in Fig. 1c.

Schematically the process is shown in Fig. 1d, where the QL terrace consisting of the Bi (red) and Te/Se layers (blue) transforms to the QL and Bi-bilayer terraces. These findings can be verified by the XPS measurements of Bi 5d core levels, shown in Fig. 1e. The spectrum for the pristine sample consists of the only one component for each 5d peak, while after the annealing additional peaks can be seen with the 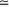
1 eV shift towards the higher BE. This peaks belong to the Bi-bilayer, in accordance with refs 13 and 32. Noteworthy, under the annealing at 400 °C one can see the complete destroying of the first surface QL with the Bi_2_ terraces formation and the appearance of the Bi_2_ areas at the surface of second QL (see the profile in Fig. 1f).

**Figure 2:**
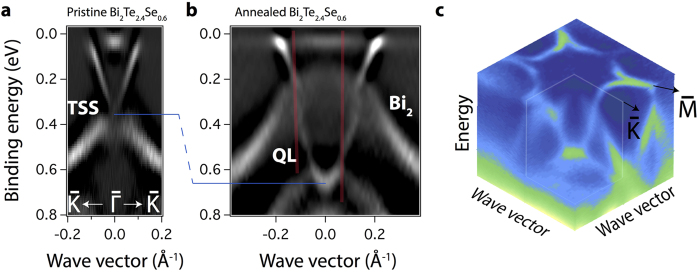
(**a**) ARPES images of the pristine cleaved and (**b**) 400 °C annealed Bi_2_Te_2.4_Se_0.6_ sample. Data collected using a photon energy of 17 eV and a linear polarization of light. The second derivatives of *N(E*) are presented for the better visualization of the states dispersion. The red lines present the emission angles, which were used for spin-resolved data in Fig. 3c. (**c**) Full ARPES 3D mapping in *k*-space of the 400 °C annealed sample.

Temperature-induced restructuring of the surface layers results in dramatic changes of the surface band structure. Experimental dispersion relations of the electronic states of the pristine and annealed Bi_2_Te_2.4_Se_0.6_ are shown in Fig. 2. First of all, the parabolic band with the minimum at the BE of 0.65 eV appears instead of the Dirac cone formed by TSS. Similar dispersion relations of the electronic states have been observed for the ultrathin films of 3D topological insulators^33^ and in Bi_4_Se_3_^13^, where QLs are separated by the Bi-bilayers. Such a behaviour is based on the interaction between the two surfaces of 3D TI in quantum tunneling regime^34^. In our annealed Bi_2_Te_2.4_Se_0.6_ this effect can be explained by: 1) the increase of the van-der-Waals spacing and detachment of the surface QLs from the bulk as a result of the annealing or 2) the formation of the different structural phase at the surface, similar to BiTe and Bi_4_Te_3_^30^,^31^.

Full ARPES *k*-space map of the electronic states of the annealed BTS near the 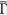 point is presented in Fig. 2c. The Fermi surface is characterized by hexagonal snowflake shape, with the bunches pointed along the 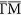 direction. Hexagonal warping of the Dirac cone, observed in a number of 3D TIs^27^,^35^ originates from the mixing of the TSS with the bulk conduction band. Owing to the high binding energy of the parabolic band minimum in our annealed system, warping leads to the huge differences of the group velocities at the Fermi level, being 4.6×10^5^ m/s for the 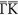 and zero for the 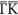 directions. This structure could give rise to such phenomena as anisotropic spin-polarized transport or density of states singularity in TIs^36^.

Besides the parabolic QLs-derived band the spectrum of the annealed Bi_2_Te_2.4_Se_0.6_ exhibits the additional broad hole-like state, dispersing from 0.6 eV at k_||_ = 0.4 Å^−1^ to 0.1 eV at the 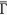 point. In accordance with refs 10, 37 and 38 this states can be related to the Bi-bilayer, formed on top of TI. Moreover, at the BE of 0.2–0.4 eV the blurring of the QL and Bi_2_ derived states dispersions is observable, that can be explained by the hybridization between them.

**Figure 3:**
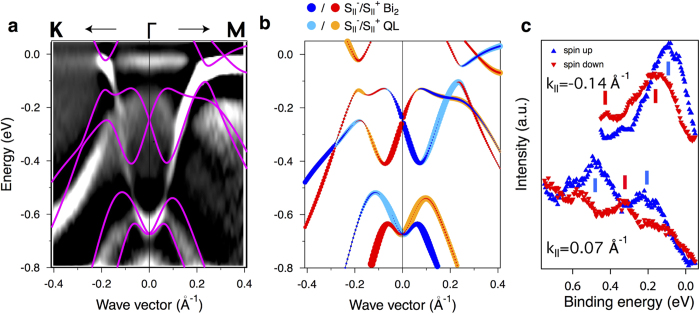
(**a**) The ARPES image of annealed Bi_2_Te_2.4_Se_0.6_ sample taken along the 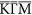 direction. The superimposed violet lines represent DFT calculated band structure of the Bi-bilayer on top of Bi_2_Te_2.4_Se_0.6_ QL. (**b**) The calculated spin resolved band structure with red (yellow) and blue (cyan) symbols corresponding to the in-plane spin polarization of the states localized at the Bi-bilayer (QL). (**c**) Spin-resolved ARPES spectra taken at the emission angles, corresponding to the red lines in Fig. 2(b), obtained using a photon energy of 17 eV.

In order to understand the peculiar behavior of the annealed TI electronic states we have performed *ab initio* calculations of the Bi-bilayer contacting with the single QL of TI. To treat the atomic structure of Bi_2_Te_2.4_Se_0.6_ Se atoms had been homogeneously distributed over three Te layers within QL in accordance with ref. 27. The calculated band dispersions along the 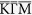 direction are superimposed on the ARPES image of the annealed sample in Fig. 3a. In accordance with the ARPES measurements one can see an electron-like band, localized in QL with the BE of 0.7 eV at the 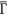 point. The Bi-bilayer states have a hole-like dispersion, and mix with the QL states at the BE region 0.2–0.4 eV.

The avoided-crossing hybridization effects between the Bi_2_ and QL states are clearly seen in the calculated band structure. In the experimental image the mixing of the states is less pronouced and the hybridization gaps are much smaller. This difference can be explained by several factors. First, the ARPES spectra include the signal from the bulk bands. The DFT calculated band structure with the inclusion of several Bi_2_Te_3_ QL is shown in Fig. 1S in Supporting Infromation. One can see that the bulk states are partially located in the region of avoided-crossing gaps and can blur the resulting experimental dispersions. The second reason of the weaker hybridization between the Bi_2_ and QL states in the experiment is the presence of the QL terraces without the Bi_2_ on top (see Fig. 1c). The photoemission spectra contain the signals from terraces both with and without Bi_2_ and the summed image appears to have a smaller mixing of the states.

The hybridization effects result in the modification of the spin structure of the system. The calculated spin resolved band structure is shown in Fig. 3b. The spin polarization of the QL state and the spin splitting of the Bi states are clearly visible. At the hybridization points, the spin-dependent avoided-crossing effects take place, leading to the modulation of the spin structure.

Figure 3c shows the spectra obtained by means of APRES with spin resolution at the emission angles, corresponding to the gray lines in Fig. 2b. One can distinguish the pronounced spin polarization of the QL and Bi states, which are inverted for the opposite wave vectors, meaning the spin-orbital character. Similar spin structure has been observed for the Bi-bilayer on top of the various substrates^12^,^39^. Meanwhile, the spin-dependent hybridization effects in the experiment are less pronounced than in Fig. 3b owing to the several reasons, noted above.

Thus, the Dirac cone in Bi_2_Te_2.4_Se_0.6_ as a result of the annealing is replaced by two hole and electron-like spin polarized bands interacting with each other. This behaviour is strongly different from those in MBE grown Bi bilayer on 3D TIs, where the Dirac cone had not been destroyed after the Bi deposition^10^,^12^,^22^,^23^,^24^. We relate the effect of parabolic band appearance instead of the Dirac cone to the detachment of the surface QL as a result of the annealing. In this case the detached QL can be considered as a 3D TI ultrathin film in quantum dimension regime. Noteworthy, after the new cleavage of annealed sample (i.e. deletion of several hundreds microns of the sample) the Dirac cone recovers meaning that the bulk atomic and electronic structure is not affected in any way.

## Conclusions

In conclusion, the electronic and spin structure of the annealed topological insulator Bi_2_Te_2.4_Se_0.6_ has been unveiled by means of spin- and angle-resolved PES. STM and XPS measurements reveal the formation of the Bi-bilayer at the surface under the annealing due to the evaporation of the Te/Se atoms from the surface QL. It has been demonstrated that the Bi-bilayer and surface QLs are characterized by the hole-like and electron-like spin-polarized states near the 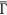 point, respectively. This 2D/3D topological insulators junction can be utilized for device fabrication purposes, such as topological edge-mode transistors or topological p-n junctions. Relatively easy formation way allows to create locally the regions with 2D TI on the surface of 3D TI using controlled annealing, for example by applying tip or laser.

## Methods

The *n*-doping samples with stoichiometry of Bi_2_Te_2.4_Se_0.6_ were grown from a presynthesized mixture of Bi_2_Te_3_ and Bi_2_Se_3_ by a modified vertical Bridgman method^40^. Monocrystals had been cleaved in ultrahigh vacuum (UHV) conditions (base pressure 1 × 10^−10^ mbar) before the measurements. The annealing process had been carried out *in situ* in UHV conditions during 30 min. The crystal quality and surface cleanliness had been verified by low energy electron diffraction and x-ray photoelectron spectroscopy. The experiments were carried out at Helmholtz-Zentrum Berlin (BESSY II) at beamlines UE112-SGM and U125/2-SGM with linearly polarized light with using Scienta R4000 energy analyzer and Mott spin detector operated at 26 keV. The angle of light incidence on the sample under normal emission was 45° relative the surface normal. The diameter of the light spot on the sample was about 50 *μ*m. STM and preliminary ARPES measurements have been carried out in Resource Center of St.-Petersburg State University “Physical methods of surface investigations”. Several tungsten tips for STM measurements were examined with the scanning electron microscope and then a sharp tip was selected and prepared with a focused ion beam at the Interdisciplinary Center for Nanotechnology of Research park of Saint Petersburg State University.

The electronic band calculations for Bi-bilayer ontop of Bi_2_Te_2.4_Se_0.6_ quantuple layer (Fig. 3a) are performed within the density functional theory (DFT) formalism with the generalized gradient approximation (GGA) of Perdew, Burke, and Ernzerhof (PBE)^41^ to the exchange correlation (XC) potential. We used Hartwigsen-Goedecker-Hutter (HGH)^42^ relativistic norm-conserving pseudopotentials which include the spin-orbit interaction (SOI). To treat the disordered Bi_2_Te_2.4_Se_0.6_ we employ a virtual crystal approximation (VCA) as implemented in the abinit code^43^, where the configuration averaged potential of a “gray” atom occupying a site in the Te-Se sublattice is defined as a mixture *V*_VCA_ = *xV*_Te_ + (1 − *x)V*_Se_ of Te (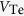) and Se (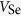) pseudopotentials.

## Additional Information

**How to cite this article:** Klimovskikh, I. I. *et al*. Spin-resolved band structure of heterojunction Bi-bilayer/3D topological insulator in the quantum dimension regime in annealed Bi_2_Te_2.4_Se_0.6_. *Sci. Rep.* **7**, 45797; doi: 10.1038/srep45797 (2017).

**Publisher's note:** Springer Nature remains neutral with regard to jurisdictional claims in published maps and institutional affiliations.

## References

[b1] Hasan M. Z. & Kane C. L. *Colloquium* : Topological insulators. Rev. Mod. Phys. 82, 3045–3067 (2010).

[b2] Qi X.-L. & Zhang S.-C. Topological insulators and superconductors. Rev. Mod. Phys. 83, 1057–1110 (2011).

[b3] Yan Y. . Topological surface state enhanced photothermoelectric effect in Bi_2_Se_3_ nanoribbons. Nano Letters 14, 4389–4394 (2014). 25046135 10.1021/nl501276e

[b4] Yan Y. . High-mobility Bi_2_Se_3_ nanoplates manifesting quantum oscillations of surface states in the sidewalls. Scientific Reports 4, 3817 EP – (2014). 24448629 10.1038/srep03817 PMC3898052

[b5] Yan Y. . Synthesis and quantum transport properties of Bi_2_Se_3_ topological insulator nanostructures. Scientific Reports 3, 1264 EP – (2013). 23405278 10.1038/srep01264 PMC3569629

[b6] Shikin A. M. . Out-of-plane polarization induced in magnetically-doped topological insulator Bi_1.37_V_0.03_Sb_0.6_Te_2_Se by circularly polarized synchrotron radiation above a curie temperature. Applied Physics Letters 109, 222404 (2016).

[b7] Shikin A. M. . Surface spin-polarized currents generated in topological insulators by circularly polarized synchrotron radiation and their photoelectron spectroscopy indication. Physics of the Solid State 58, 1675–1686 (2016).

[b8] König M. . Quantum spin hall insulator state in HgTe quantum wells. Science 318, 766–770 (2007). 17885096 10.1126/science.1148047

[b9] Kane C. L. & Mele E. J. Z_2_ topological order and the quantum spin hall effect. Phys. Rev. Lett. 95, 146802 (2005). 16241681 10.1103/PhysRevLett.95.146802

[b10] Hirahara T. . Interfacing 2D and 3D topological insulators: Bi(111) bilayer on Bi_2_Te_3_. Phys. Rev. Lett. 107, 166801 (2011). 22107414 10.1103/PhysRevLett.107.166801

[b11] Lee P. . Topological modification of the electronic structure by Bi-bilayers lying deep inside bulk Bi_2_Se_3_. Journal of Physics: Condensed Matter 28, 085002 (2016). 26852742 10.1088/0953-8984/28/8/085002

[b12] Yeom H. W. . Transforming a surface state of a topological insulator by a Bi capping layer. Phys. Rev. B 90, 235401 (2014).

[b13] Valla T. . Topological semimetal in a Bi-Bi_2_Se_3_ infinitely adaptive superlattice phase. Phys. Rev. B 86, 241101 (2012).

[b14] Shokri R. . Atomic and electronic structure of bismuth-bilayer-terminated Bi_2_Se_3_ (0001) prepared by atomic hydrogen etching. Phys. Rev. B 91, 205430 (2015).

[b15] Zhang L., Yan Y., Wu H.-C., Yu D. & Liao Z.-M. Gate-tunable tunneling resistance in graphene/topological insulator vertical junctions. ACS Nano 10, 3816–3822 (2016). 26930548 10.1021/acsnano.6b00659

[b16] Zhang J., Triola C. & Rossi E. Proximity effect in graphene/topological-insulator heterostructures. Phys. Rev. Lett. 112, 096802 (2014). 24655268 10.1103/PhysRevLett.112.096802

[b17] Bian G. . Experimental observation of two massless Dirac-fermion gases in graphene-topological insulator heterostructure. 2D Materials 3, 021009 (2016).

[b18] Klimovskikh I. I. . Spin–orbit coupling induced gap in graphene on Pt(111) with intercalated Pb monolayer. ACS Nano 11, 368–374 (2017). 28005333 10.1021/acsnano.6b05982

[b19] Drozdov I. K. . One-dimensional topological edge states of bismuth bilayers. Nat Phys 10, 664–669 (2014).

[b20] Wada M., Murakami S., Freimuth F. & Bihlmayer G. Localized edge states in two-dimensional topological insulators: Ultrathin Bi films. Phys. Rev. B 83, 121310 (2011).

[b21] Bian G. . Engineering electronic structure of a two-dimensional topological insulator Bi(111) bilayer on Sb nanofilms by quantum confinement effect. ACS Nano 10, 3859–3864 (2016). 26932368 10.1021/acsnano.6b00987

[b22] Eich A. . Intra- and interband electron scattering in a hybrid topological insulator: Bismuth bilayer on Bi_2_Se_3_. Phys. Rev. B 90, 155414 (2014).

[b23] Kim S. H. . Edge and interfacial states in a two-dimensional topological insulator: Bi(111) bilayer on Bi_2_Te_2_Se. Phys. Rev. B 89, 155436 (2014).

[b24] Lei T. . Electronic structure evolution of single bilayer Bi(111) film on 3D topological insulator Bi_2_Te_x_Se_3−x_ surfaces. Journal of Physics: Condensed Matter 28, 255501 (2016). 27166645 10.1088/0953-8984/28/25/255501

[b25] Coelho P. M. . Temperature-induced coexistence of a conducting bilayer and the bulk-terminated surface of the topological insulator Bi_2_Te_3_. Nano Letters 13, 4517–4521 (2013). 23952071 10.1021/nl402450b

[b26] Schouteden K. . Annealing-induced bi bilayer on Bi_2_Te_3_ investigated via quasi-particle-interference mapping. ACS Nano 10, 8778–8787 (2016). 27584869 10.1021/acsnano.6b04508

[b27] Shikin A. M. . Electronic and spin structure of the topological insulator Bi_2_Te_2.4_Se_0.6_. Phys. Rev. B 89, 125416 (2014).

[b28] Filyanina M. V. . Specific features of the electronic, spin, and atomic structures of a topological insulator Bi_2_Te_2.4_Se_0.6_. Physics of the Solid State 58, 779 (2016).

[b29] He X. . Surface termination of cleaved Bi_2_Se_3_ investigated by low energy ion scattering. Phys. Rev. Lett. 110, 156101 (2013). 25167285 10.1103/PhysRevLett.110.156101

[b30] Bos J. W. G., Zandbergen H. W., Lee M.-H., Ong N. P. & Cava R. J. Structures and thermoelectric properties of the infinitely adaptive series (Bi_2_)_*m*_(Bi_2_Te_3_)_*n*_. Phys. Rev. B 75, 195203 (2007).

[b31] Lind H. & Lidin S. A general structure model for Bi–Se phases using a superspace formalism. Solid State Sciences 5, 47–57 (2003).

[b32] Gibson Q. D. . Termination-dependent topological surface states of the natural superlattice phase Bi_4_Se_3_. Phys. Rev. B 88, 081108 (2013).

[b33] Neupane M. . Observation of quantum-tunnelling-modulated spin texture in ultrathin topological insulator Bi_2_Se_3_ films. Nature Comm. 5, 3841 (2014). 10.1038/ncomms4841 24815418

[b34] Landolt G. . Spin texture of Bi_2_Se_3_ thin films in the quantum tunneling limit. Phys. Rev. Lett. 112, 057601 (2014). 24580629 10.1103/PhysRevLett.112.057601

[b35] Chen Y. L. . Experimental realization of a three-dimensional topological insulator, Bi_2_Se_3_. Science 325, 178–181 (2009). 10.1126/science.1173034 19520912

[b36] Repin E. & Burmistrov I. Surface states in a 3D topological insulator: the role of hexagonal warping and curvature. ZhETF 148, 584 (2015).

[b37] Ast C. R. & Höchst H. Electronic structure of a bismuth bilayer. Phys. Rev. B 67, 113102 (2003).

[b38] Koroteev Y. M., Bihlmayer G., Chulkov E. V. & Blügel S. First-principles investigation of structural and electronic properties of ultrathin Bi films. Phys. Rev. B 77, 045428 (2008).

[b39] Klimovskih I. I. . Spin polarization of quantum-well and interface states of ultrathin films of Bi on W(110) with ag interlayers. Bulletin of the Russian Academy of Sciences: Physics 78, 39–42 (2014).

[b40] Kokh K. A., Nenashev B. G., Kokh A. E. & Shvedenkov G. Y. Application of a rotating heat field in bridgman–stockbarger crystal growth. Journal of Crystal Growth 275, e2129–e2134 (2005).

[b41] Perdew J. P., Burke K. & Ernzerhof M. Generalized gradient approximation made simple. Phys. Rev. Lett. 77, 3865–3868 (1996). 10062328 10.1103/PhysRevLett.77.3865

[b42] Hartwigsen C., Goedecker S. & Hutter J. Relativistic separable dual-space gaussian pseudopotentials from H to RN. Phys. Rev. B 58, 3641–3662 (1998). 10.1103/physrevb.54.1703 9986014

[b43] Gonze X. . Abinit: First-principles approach to material and nanosystem properties. Computer Physics Communications 180, 2582–2615 (2009).

